# DEK48 Is Required for RNA Editing at Multiple Mitochondrial Sites and Seed Development in Maize

**DOI:** 10.3390/ijms23063064

**Published:** 2022-03-12

**Authors:** Dalin Yang, Shi-Kai Cao, Huanhuan Yang, Rui Liu, Feng Sun, Le Wang, Miaodi Wang, Bao-Cai Tan

**Affiliations:** Key Laboratory of Plant Development and Environment Adaptation Biology, Ministry of Education, School of Life Sciences, Shandong University, Qingdao 266237, China; yungshuyezi@163.com (D.Y.); caoshk5233@163.com (S.-K.C.); yanghuanhuan7@163.com (H.Y.); liuxiaoshuang_6@163.com (R.L.); epusun@sdu.edu.cn (F.S.); 17862979051@163.com (L.W.); wmd963359448@163.com (M.W.)

**Keywords:** PPR-DYW protein, seed development, mitochondria, complex I, RNA editing

## Abstract

In flowering plants, C-to-U RNA editing can be critical to normal functions of mitochondrion-encoded proteins. Mitochondrial C-to-U RNA editing is facilitated by many factors from diverse protein families, of which the pentatricopeptide repeat (PPR) proteins play an important role. Owing to their large number and frequent embryo lethality in mutants, functions of many PPRs remain unknown. In this study, we characterized a mitochondrion-localized DYW-type PPR protein, DEK48, functioning in the C-to-U RNA editing at multiple mitochondrial transcripts in maize. Null mutation of *Dek48* severely arrests embryo and endosperm development, causing a *defective kernel* (*dek*) phenotype, named *dek48*. DEK48 loss of function abolishes the C-to-U editing at *nad3*-185, -215, and *nad4*-376, -977 sites and decreases the editing at 11 other sites, resulting in the alteration of the corresponding amino acids. Consequently, the absence of editing caused reduced assembly and activity of complex I in *dek48*. Interestingly, we identified a point mutation in *dek48-3* causing a deletion of the Tryptophan (W) residue in the DYW motif that abolishes the editing function. In sum, this study reveals the function of DEK48 in the C-to-U editing in mitochondrial transcripts and seed development in maize, and it demonstrates a critical role of the W residue in the DYW triplet motif of DEK48 for the C-to-U editing function in vivo.

## 1. Introduction

Mitochondria are highly metabolically active organelles, producing ATP for eukaryotic cell activities through the electron transport respiratory chain (ETC). The core components of ETC consist of five respiratory complexes: I–V. Electrons from nicotinamide adenine dinucleotide (NADH) dehydrogenase (complex I) and succinate dehydrogenase (complex II) are transported through ubiquinone and cytochrome *c* reductase (complex III) to cytochrome *c* oxidase (complex IV), finally generating most cellular ATP by ATP synthase (complex V) [1]. During evolution, approximately 95% of the ancestral mitochondrial genes from the α-proteobacteria were lost or transferred to host nucleus, and only 5% were retained in the mitochondrial genome [2]. The expression of these mitochondrial genes is highly regulated by numerous nucleus-encoded factors including transcription, post-transcriptional processing, and translation. A major regulatory process is RNA processing, which includes intron splicing, RNA cleavage, RNA maturation and stabilization, and RNA editing [3].

RNA editing alters transcript sequences through insertion, deletion, or conversion of nucleotide, resulting in changes to the genetic information encoded by the DNA sequences [4]. In land plant plastids and mitochondria, conversion of cytidine (C) to uridine (U) is the main type of RNA editing. In plastids, 20–40 C-to-U editing sites are identified, whereas over 400 sites exist in mitochondria [5]. Editing occurs in all RNAs, including mRNAs, tRNAs, and rRNAs, and in all regions, including UTRs, exons, and introns. Editing is considered a mechanism to remedy genetic mutations incurred in DNA to ensure the coding of functional proteins or RNAs [6]. C-to-U editing is essential to the function of plastids and mitochondria, as it is important for the organelle encoded proteins. As such, defects in editing lead to severe consequences in plant growth and development, including embryo lethality [7,8,9]. For example, defective editing of mitochondrial *nad7* at specific sites leads to slow growth in *Arabidopsis* and arrests the embryo and endosperm development in maize [10,11,12].

Several families of nucleus-encoded factors are found to function in RNA editing, including RNA editing factor interacting proteins (RIPs)/multiple organellar RNA editing factor (MORF) [13,14], organelle RNA recognition motif (ORRM) proteins [15,16,17], organelle zinc-finger 1 (OZ1) [18], RNA helicase [19], protoporphyrinogen IX oxidase [20], and PPR proteins [21]. PPR proteins are sequence-specific RNA-binding proteins existing prevalently in land plants [22]. Based on domain constitution, PPR proteins are classified into two subclasses, P and PLS. The P subclass contains a canonical P motif, whereas the PLS subclass consists of P, L, and S motifs and additional C-terminal domains (E1, E2, E+, and DYW) [22,23]. Most P-class PPR proteins are reported to function on translation initiation, RNA maturation, RNA stability, and intron splicing, whereas PLS-type PPR proteins are mostly involved in C-to-U RNA editing [21]. The chemical nature of C-to-U editing involves the deamination of cytidine, and the DYW domain has been proved to possess cytidine deaminase activity [24,25,26].

More than 500 PPRs are found in the maize genome, and 82 belong to the PPR-DYW subclass [27,28]. Nine PPR-DYW proteins have been characterized in maize thus far. A DYW-subgroup PPR protein, PPR2263, is specifically responsible for the editing of mitochondrial *nad5* and *cob* transcript. The mutation of *PPR2263* results in growth defects in kernels and seedlings [9]. DYW-type PPR proteins *qKW9* and *ZmPPR26* are involved in C-to-U RNA editing at *ndhB*-737 and *atpA*-1148 sites in chloroplast, respectively. Loss of *qKW9* and *ZmPPR26* affects photosynthesis, causing small kernel and albino seedling lethality, respectively [29,30]. Moreover, PPR-DYW proteins EMP5, EMP17, EMP18, EMP21, PPR27, and DEK46 are all required for C-to-U RNA editing at multiple mitochondrial transcripts and critical to the mitochondrial functions and seed development [7,8,12,31,32,33]. Loss of these PPR-DYW proteins impairs C-to-U RNA editing, disrupts the normal organellar function, and affects seed development and plant growth, suggesting the essential roles of PPR-DYWs in organellar RNA metabolism and plant development.

In this study, we characterized a mitochondrion-localized DYW-subgroup PPR protein, DEK48, which is required for the C-to-U editing of multiple mitochondrial transcripts in maize. Loss of DEK48 function abolishes C-to-U editing at *nad3*-185, 215 and *nad4*-376, 977 sites and reduces the editing at eleven sites, resulting in a reduced assembly of mitochondrial complex I and severely arrested kernel development. In sum, this study reveals that PPR-DYW protein DEK48 plays a vital role in RNA editing, mitochondrial function, and seed development in maize. Furthermore, through molecular analysis of a mutant with a deletion of the W (tryptophan) residue in the DYW triplet, this study demonstrates that this W is essential to the editing function of DEK48.

## 2. Results

### 2.1. DEK48 Is a Canonical DYW-Subgroup PPR Protein

**Figure 1:**
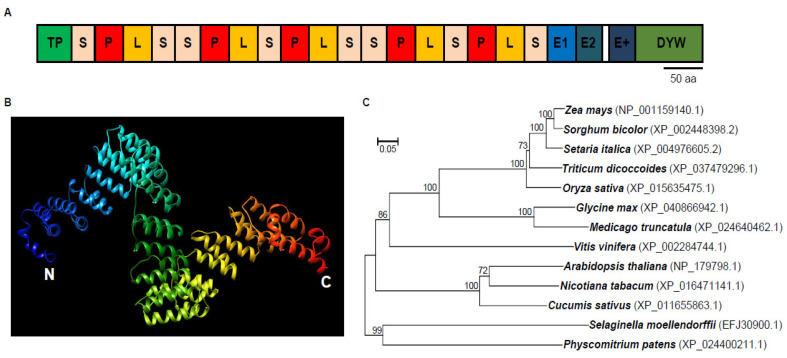
*Dek48* encodes a canonical DYW-type PPR protein. (**A**) The predicted DEK48 contains 18 PPR motifs, an E1/E2, an E+ domain, and a DYW domain. TP; targeted peptide. aa; amino acid. (**B**) Predicted structure of DEK48 protein. Blue alpha helices indicate the N-terminus and red helices indicate the C-terminus of DEK48. (**C**) Phylogenic analysis of DEK48 proteins in representative species.

*Dek48* (*GRMZM2G017197*) is an intronless gene and encodes a PLS-type PPR protein with 18 PPR motifs, an E1/E2, an E+ domain, and a DYW domain at the C-terminus, indicating that DEK48 is a canonical PPR-DYW protein (Figure 1A). The model of DEK48 structure was predicted based on template PPR10 using Phyre2 (http://www.sbg.bio.ic.ac.uk/phyre2/html/page.cgi?id=index (accessed on 1 November 2018)). As shown in Figure 1B, DEK48 has multiple paired antiparallel alpha helices forming a super helix. Phylogenetic analysis revealed that orthologous proteins of DEK48 are found among different plant species (Figure 1C), suggesting an extensive conservation in the protein sequences from *Physcomitrium patens* to mono- and eudicotyledonous species. These orthologous proteins of DEK48 have not been analyzed.

### 2.2. Phenotypic and Genetic Characterization of dek48-1

**Figure 2:**
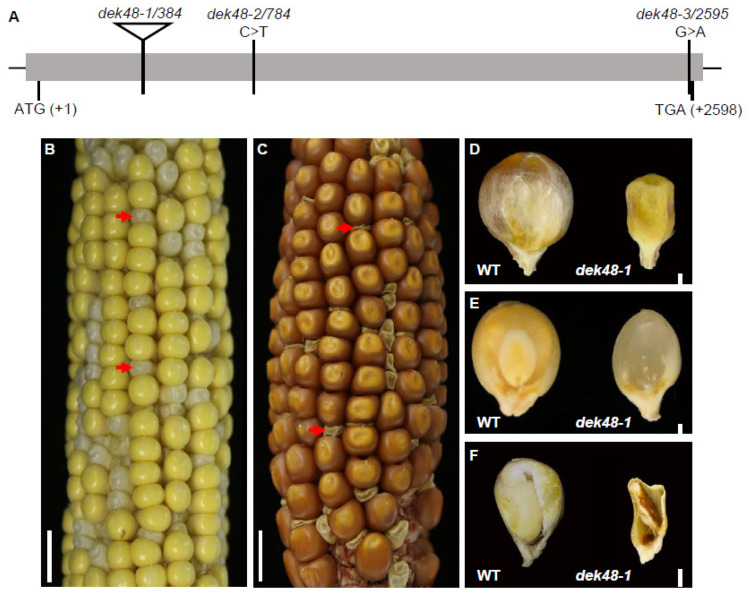
Phenotypic characterization of *dek48-1* kernels. (**A**) Schematic structure of the *Dek48* gene and positions of *Mutator* (*Mu*) insertions. The *Mu* insertion site of *dek48-1* and point mutation sites of *dek48-2* and *dek48-3* are shown in detail. (**B**) A segregating ear of *dek48-1*/+ maize at 12 days after pollination (DAP). Arrows point to *dek48-1* mutant kernels. (**C**) Mature ear of self-pollinated *dek48-1* heterozygotes. Arrows point to *dek48-1* mutant kernels. (**D**) Embryo side of mature kernels of wild-type (WT) and *dek48-1* kernels. (**E**) The embryo and endosperm of WT and *dek48-1* kernels at 12 DAP. (**F**) Dissection of mature WT and *dek48-1* kernels along the embryo axis. Scale bars = 1 cm in (**B**,**C**); 1 mm in (**D**–**F**).

To reveal the function of DEK48, a *Mutator* (*Mu*) insertional mutant (*dek48-1*) was isolated from the UniformMu population [34]. Sequencing results confirm the *Mu* insertion at 384 bp downstream from the ATG in *dek48-1* (Figure 2A). The self-pollinated *dek48-1*/+ heterozygotes segregated *defective kernels* (*dek*) and wild-type (WT) at a 1:3 ratio (*dek48-1*: WT, 132: 404, *p* < 0.05), suggesting a monogenic recessive mutation (Figure 2B,C). Compared with the WT, the *dek48-1* kernels are smaller at maturity (Figure 2D). Sectioning of the *dek48-1* and the WT sibling kernels showed that the embryo and endosperm development were severely defective in *dek48-1* (Figure 2E,F). Linkage analysis in a population of 36 plants by genomic PCR with *Mu*-TIR8 primer and *Dek48*-specific primers showed that the *dek* phenotype is tightly linked with the *Mu* insertion (Figure S1). Wildtype *Dek48* transcripts could not be detected in *dek48-1* by RT-PCR (Figure S2), indicating a probably null mutation.

To confirm that *GRMZM2G017197* is the causal gene for the *dek* phenotype, two additional alleles were isolated, *dek48-2* and *dek48-3*, from the maize ethyl methane sulfonate (EMS)-mutagenized population [35]. As indicated in Figure 2A, *dek48-2* contains a mutation of C-to-T at +784 bp, resulting in a stop codon (from CAA to TAA) that leads to a truncation of 605 amino acid residues in the C-terminus of DEK48. Similarly, *dek48-3* carries a mutation of G-to-A at + 2595 bp, causing a stop codon (TGG to TGA) that generates a loss of only the last tryptophan (W) residue in the DYW triplet of DEK48. Genetic analysis revealed that *dek48-2* displayed the *dek* phenotype (Figure S3A) and *dek48-3* displayed a *small kernel* (*smk*) phenotype (Figure S3B). Heterozygous *dek48-2*/+ and *dek48-3*/+ were crossed with *dek48-1*/+ heterozygous, respectively. The cross progenies produced approximately 25% *dek* kernels in the cross *dek48-1*/+ X *dek48-2/+* (Figure S3C) and approximately 25% *smk* kernels in the cross *dek48-1*/+ X *dek48-3/+* (Figure S3D). These results indicate that *GRMZM2G017197* is the causal gene for the *dek48* phenotype.

**Figure 3:**
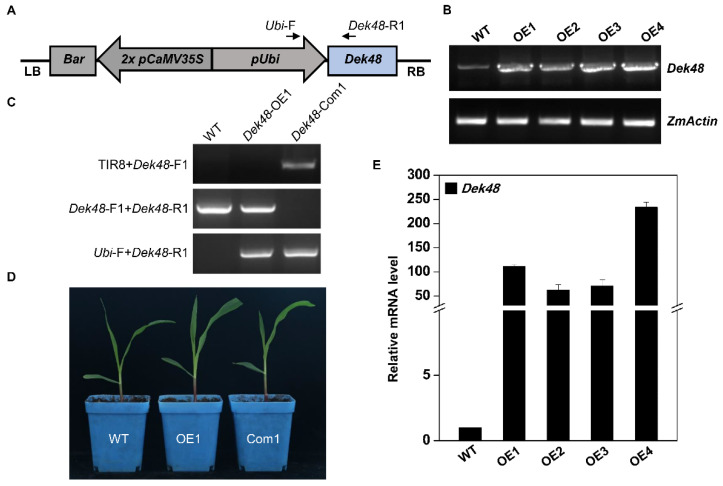
Over-expression of *Dek48* causes the embryo-lethality of *dek48*. (**A**) Transgenic construct of overexpression vector containing *Dek48*. (**B**) Semi-quantitative RT-PCR analysis of *Dek48* transcription profiling in WT and *Dek48* overexpression plants. The expression levels were normalized to *ZmActin* (*GRMZM2G126010*). (**C**) Genotyping of the overexpressed lines *Dek48*-OE1 and the complemented lines *Dek48*-Com1 harboring transgene *Dek48* expression and *Mu* insertion in the endogenous *Dek48*. (**D**) Seedling phenotype comparison among overexpressed lines *Dek48*-OE1, complemented lines *Dek48*-Com1, and wide type. (**E**) Quantitative RT-PCR analysis of *Dek48* transcription profiling in WT and *Dek48* overexpressed plants. The expression levels were normalized to *ZmActin* (*GRMZM2G126010*). Data are means (±SE) of three biological replicates.

To further confirm the *dek48* phenotype is caused by the disruption of *GRMZM2G017197*, we created transgenic lines overexpressing *Dek48*. The full-length ORF of *Dek48* was cloned into the pUNTF binary vector under the maize *ubiquitin1* promoter (Figure 3A). The overexpression of transgenic plants (*Dek48*-OE) was obtained via *Agrobacterium tumefaciens* transformation of the KN5585 inbred line. Four independent lines (*Dek48*-OE1 to OE4) were generated. RT-PCR and qRT-PCR results show a high level of *Dek48* expression in these transgenic plants compared with WT (Figure 3B,E). We crossed *Dek48*-OE1 with *dek48-1*/+ heterozygotes to generate the F1 progenies. The plants containing *Dek48*-OE1 transgene and *Mu* insertion were selected and self-pollinated. Homozygous for *dek48* harboring, the *Dek48* transgene was identified in the seedlings of F2 progeny, indicating that *Dek48*-OE1 rescues the embryo lethal phenotype of *dek48-1* (Figure 3C,D). These results confirm that the *dek48* phenotype is caused by mutation of the *GRMZM2G017197* gene, hereafter referred to as *Dek48*.

### 2.3. Embryo and Endosperm Development Are Arrested in dek48-1

**Figure 4:**
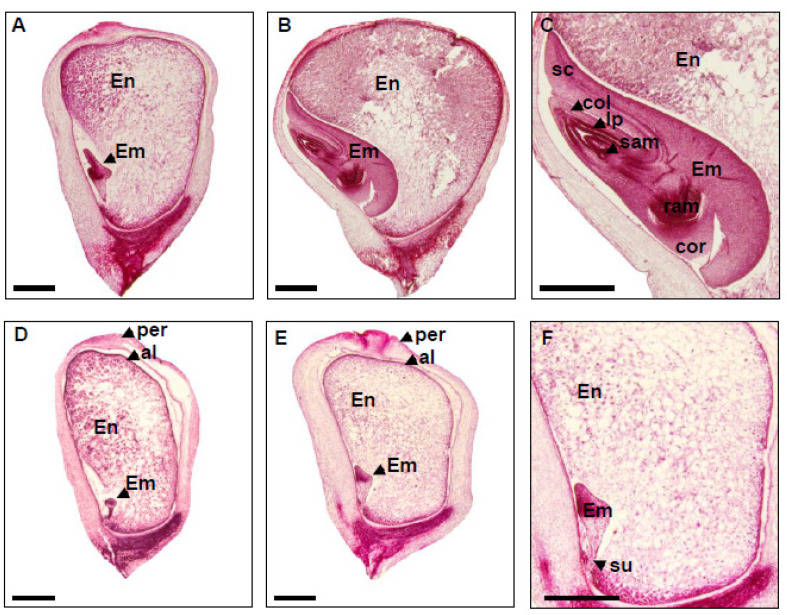
Embryo and endosperm development are arrested in the *dek48* mutant. (**A**–**F**) Paraffin sections of wild-type and *dek48-1* kernels at 9 DAP and 15 DAP. WT kernels at 9 DAP (**A**) and 15 DAP (**B**,**C**); *dek48-1* kernels at 9 DAP (**D**) and 15 DAP (**E**,**F**). (**C**,**F**) are enlarged views of the embryo in (**B**,**E**), respectively. Em, embryo; En, endosperm; sc, scutellum; col, coleoptile; lp, leaf primordial; per, pericarp; al, aleurone; su, suspensor; cor, coleorhizae; sam, shoot apical meristem; ram, root apical meristem. Scale bars = 1 mm in (**A**,**B**,**D**,**E**); 0.5 mm in (**C**,**F**).

The developmental stages of maize embryo consist of transition, coleoptilar, and late embryogenesis. Meanwhile, endosperm development includes coenocytic, cellularization, differentiation, and maturation stages [36]. To pinpoint the severe arrest of embryo and endosperm development in *dek48-1*, the mutant and WT kernels from the same segregating ear were sectioned and observed under light microscopy (Figure 4). At 9 DAP, the WT embryo developed well, reaching the coleoptilar stage (Figure 4A), whereas the *dek48-1* embryo was inhibited at the transition stage (Figure 4D). At 15 DAP, the WT embryo continued to develop, reaching the maturation stage as indicated by the differentiated leaf primordia (LP), shoot apical meristem (SAM), and root apical meristem (RAM) (Figure 4B,C). In contrast, *dek48-1* embryo displayed no clear differentiation except some densely packed cells on top of the embryo proper, suggesting an arrest at the transition stage (Figure 4E,F). Similarly, at 9 and 15 DAP, the endosperm development was severely delayed in *dek48-1* compared with the WT (Figure 4). These results indicate that loss of function in *Dek48* arrests the embryo development at the transition stage and severely delays the endosperm development. As a result, the *dek48* alleles are embryo-lethal, implying that DEK48 plays an essential role in embryogenesis and endosperm development in maize.

### 2.4. DEK48 Is Targeted to Mitochondria

**Figure 5:**
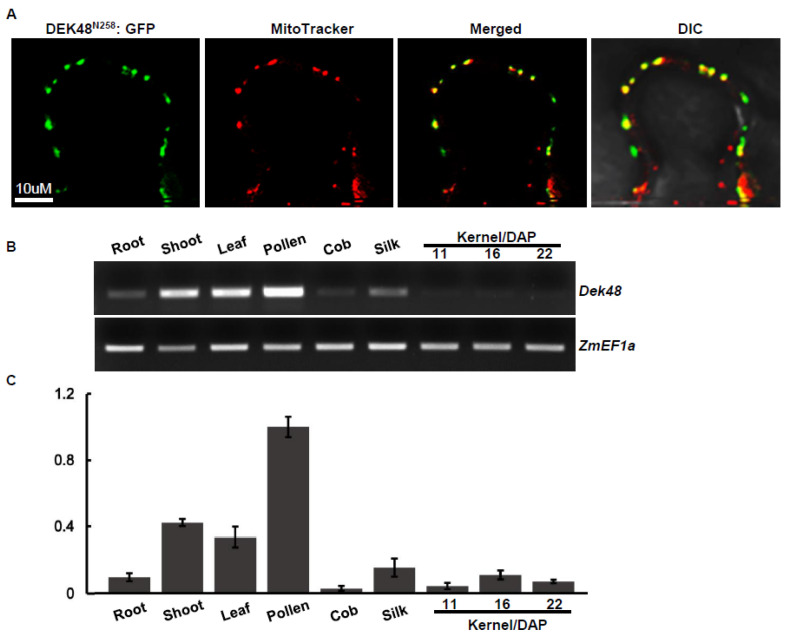
DEK48 is targeted to mitochondria. (**A**) Subcellular localization of DEK48. A DEK48^N258^-GFP fusion protein was transiently expressed in tobacco epidermal cells. MitoTracker Red was used as a mitochondrial marker. DIC, differential interference contrast; N258, the N terminus 258 amino acids of DEK48. Scale bars = 10 μm. (**B**) Semi-quantitative RT-PCR analysis of *Dek48* expression in WT tissues and developing kernels. Normalization was performed against *ZmEF1α* (*GRMZM2G153541*). (**C**) Quantitative RT-PCR analysis of *Dek48* expression in WT tissues and developing kernels. Normalization was performed against *ZmEF1α* (*GRMZM2G153541*). Data are means (±SE) of three biological replicates.

To determine the subcellular localization, we fused the full-length DEK48 with green fluorescent protein (GFP) at an N–C orientation under the control of the 35S CaMV promoter in the pGWB5 vector. The fusion was transiently expressed in tobacco epidermal cells using the *Agrobacterium* infiltration. No signals were detected. We speculate that this might be due to poor expression of the full-length DEK48, as it was frequently detected in such proteins [8,37]. Then, we fused the N-terminal 258 amino acids of DEK48 to GFP in the pGWB5 vector to generate the DEK48^N258^-GFP fusion. When expressed in tobacco cells, strong green fluorescence signals were detected in dots that were merged with the red fluorescence signals of mitochondria marked by the MitoTracker (Figure 5A). This result indicates that DEK48 is targeted to mitochondria.

RT-PCR and qRT-PCR analysis showed that *Dek48* is ubiquitously expressed in various maize tissues and developing kernels, with relatively high expression in pollen and low expression in cob and developing kernels (Figure 5B,C), suggesting that *Dek48* is a constitutive gene and may play an important role in all tissues during maize growth and development.

### 2.5. DEK48 Is Required for the C-to-U RNA Editing at Multiple Mitochondrial Sites

**Figure 6:**
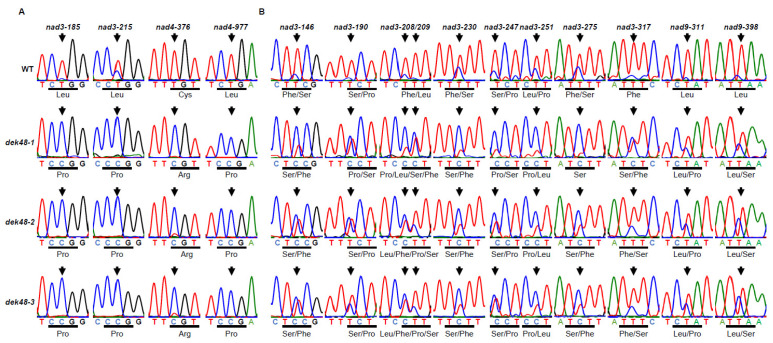
RNA editing defects of mitochondrial genes in *dek48* alleles. (**A**) Loss of DEK48 function abolishes the editing at *nad3*-185, -215 and *nad4*-376, -977 sites. Analysis of RNA editing at *nad3*-185, -215 and *nad4*-376, -977 sites in the transcripts from embryo and endosperm of WT and *dek48* mutants at 12 DAP. (**B**) Decreased RNA editing in *dek48* alleles relative to WT at eleven other sites. Editing sites are indicated by arrows. Codons containing the edited nucleotide are underlined, and the coded amino acids are shown.

Previous studies showed that most PPR-DYW proteins are involved in C-to-U editing in plant organelles [7,38,39]. To uncover the function of DEK48, we analyzed the transcripts of 35 mitochondrion-encoded genes between WT and *dek48-1* by RT-PCR. The nearly full-length cDNAs were amplified from WT and *dek48-1* kernels in the same ear at 12 DAP. We analyzed the editing efficiency by direct sequencing of these PCR amplicons. Results show that the C-to-U editing at the *nad3*-185, -215 and *nad4*-376, -977 sites was completely abolished (Figure 6A), and the editing at eleven other sites (*nad3*-146, -190, -208, -209, -230, -247, -251, -275, -317 and *nad9*-311, -398) was substantially decreased in *dek48-1* compared with in WT (Figure 6B). Analysis of *dek48-2* and *dek48-3* showed similar results at all fifteen sites, except that *dek48-1* is more severe.

**Figure 7:**
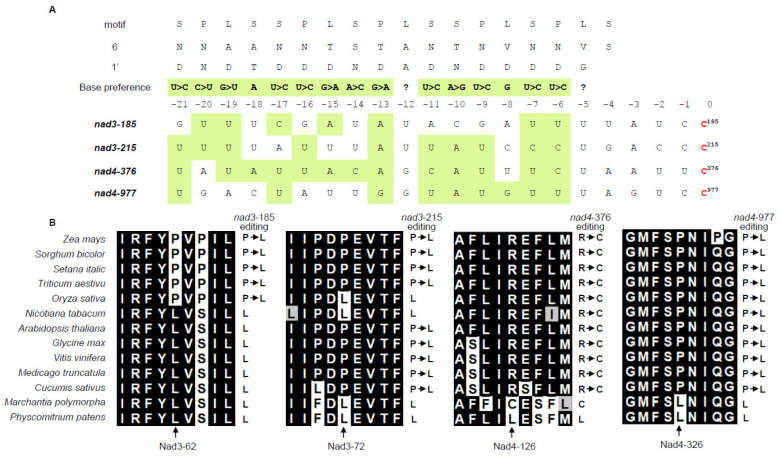
The Nad3 Leu^62^ and Nad4 Cys^126^ and Leu^326^ residues are highly conserved, while the residue at Nad3 Leu^72^ is variable. (**A**) Alignment of the amino acid residues at position 6 and 1′ in each PPR motif of the DEK48 protein on the putative respective recognition sites in *nad3* and *nad4* mRNA based on the recognition code referred to by Barkan et al., 2012; Takenaka et al., 2013. Nucleotides matching the amino acid combination are indicated in light green. (**B**) Alignment of the amino acid residues encoded by *nad3*-185, -215, and *nad4*-376, -977 in multiple species. The protein sequences are derived from mitochondrial gDNA in GenBank/EMBL databases.

PPR proteins bind to RNA substrate in a sequence-specific manner, where target sequence is recognized by amino acid residue combination at the sixth and next 1′ position of the PPR motif [40,41]. Based on this code, potential binding sites of DEK48 were predicted. Results show that the nucleotides upstream of *nad3*-185, -215 and *nad4*-376, -977 sites are well-aligned to the combinatorial codes (Figure 7A). The abolished editing in *dek48-1* alters the encoded amino acid from Leu (CUG)^62^ to Pro (CCG)^62^ and Leu (CUG)^72^ to Pro (CCG)^72^ in *nad3*, and Cys (UGU)^126^ to Arg (CGU)^126^ and Leu (CUG)^326^ to Pro (CCG)^326^ in *nad4* (Figure 6A). Alignment of genomic DNA sequences of *nad3* and *nad4* orthologs indicated that these four amino acids were highly conserved from *Physcomitrium patens* to mono- and eudicotyledonous species (Figure 7B), implying that these residues are probably important to the function of Nad3 and Nad4.

### 2.6. Loss of Function in DEK48 Affects the Assembly and Activity of Complex I

**Figure 8:**
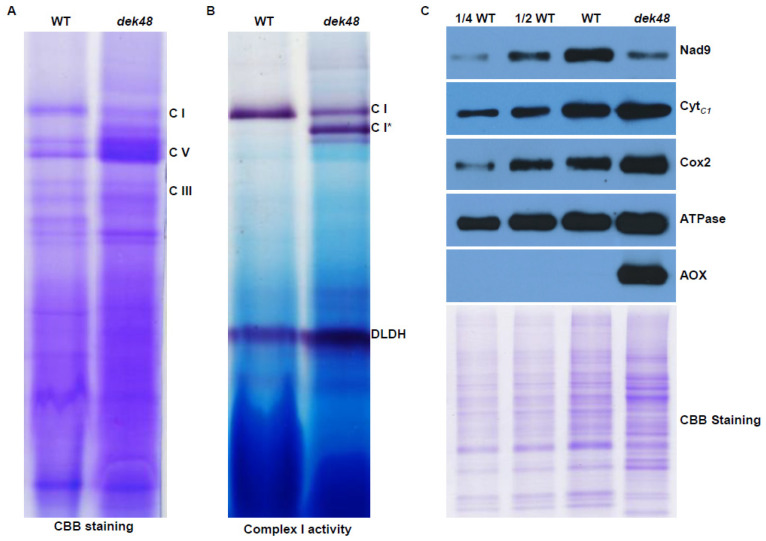
Blue native PAGE analyses of mitochondrial complexes in *dek48-1* mutant. (**A**) Blue native (BN) gel was stained with Coomassie brilliant blue (CBB). The positions of complex I, III, and V are indicated. (**B**) In-gel NADH dehydrogenase activity of complex I. The activity of dihydrolipoamide dehydrogenase (DLDH) is used as a loading control. Asterisks indicate partially assembled complex I. (**C**) Western blot analysis with antibodies against Nad9, Cyt*_C1_*, Cox2, ATPase α subunit, and AOX. CBB staining demonstrates that equal amounts of mitochondrial proteins were loaded.

Nad3 and Nad4 are the core subunits of mitochondrial complex I, an entry point of the oxidative phosphorylation (OXPHOS) pathway [42,43]. To assess the impact of the *Dek48* mutation, we analyzed the assembly of complex I and its NADH dehydrogenase activity by blue native polyacrylamide gel electrophoresis (BN-PAGE) and in-gel NADH dehydrogenase activity staining. As shown in Figure 8A, Coomassie brilliant blue (CBB) staining showed that the abundance of complex I was drastically decreased in *dek48-1* compared with the WT, whereas complex III and V were increased. The in-gel NADH dehydrogenase activity assay showed a similar decreased activity of complex I in *dek48-1* (Figure 8B). Two bands smaller than complex I were detected and showed NADH dehydrogenase activity (Figure 8B), suggesting potential blocked sub-complexes of complex I in the assembly process. These results suggest that the loss of DEK48 function inhibits the assembly of complex I. Furthermore, we employed Western blotting to examine the protein abundance of mitochondrial complexes by specific antibodies against Nad9 (a subunit of complex I), Cyt*_C1_* (a subunit of complex III), Cox2 (a subunit of complex IV), and ATPase (α subunit of complex V). Results show the abundance of Nad9 was decreased in *dek48-1*, whereas the levels of Cyt*_C1_*, Cox2, and ATPase were slightly increased (Figure 8C). This result is consistent with the BN-PAGE analysis.

### 2.7. Alternative Respiratory Pathway Is Enhanced in dek48

The dysfunction of the electron transport respiratory chain frequently enhances the alternative respiratory pathway [11,44]. To investigate whether the alternative respiratory pathway was affected in the *dek48-1* mutant, we analyzed the protein abundance of alternative oxidase (AOX) by Western blotting using specific antibody against AOX. The results show that AOX was dramatically accumulated in *dek48-1*, in contrast to no AOX being detected in WT (Figure 8C). This confirms that the block in the complex assembly leads to an enhanced expression of the alternative respiratory pathway. Maize AOX proteins were encoded by three *AOX* genes, *AOX1*, *AOX2*, and *AOX3*. RT-PCR and qRT-PCR results show that the expression level of *AOX2* was remarkably increased in *dek48-1*, whereas the *AOX1* and *AOX3* transcript levels were indistinguishable from those of the WT siblings (Figure S4). These results indicate that the alternative respiratory pathway was enhanced in *dek48*.

## 3. Discussion

### 3.1. DEK48 Is Required for C-to-U Editing, Complex I Assembly, and Kernel Development

DEK48 is a new PPR-DYW protein with unknown function. Loss of DEK48 abolished the C-to-U editing at four sites of *nad3* and *nad4* and significantly decreased the editing at nine sites of *nad3* and two sites of *nad9* (Figure 6). Prediction of the recognizing sequence of DEK48 by the PPR codes shows a high level of agreement with the 5′ sequences of the editing-abolished sites, suggesting that they can specifically recognize these editing sites [40,41] (Figure 7A). The lack of editing caused amino acid residue changes in Nad3 and Nad4. Together with other changes at other amino acid residues, these alterations impair the mitochondrial function by inhibiting the assembly and activity of complex I in *dek48* (Figure 6 and Figure 8). The block in the OXPHOS pathway leads to an elevated AOX pathway (Figure S4). Together, these data indicate that DEK48 functions in the C-to-U editing at the 15 sites in three mitochondrial gene transcripts, particularly 2 sites in *nad3* and 2 sites in *nad4* whose editing completely depends on DEK48.

It is uncommon for a PPR-DYW protein to function in the editing of more than 10 sites, and is even rarer to find a PPR-DYW protein that targets 11 sites in a single gene. Most of the reported PPR-DYW proteins have the editing function at one or a few sites. However, DEK48 functions in the editing at 11 sites in *nad3*, 2 in *nad4*, and 2 in *nad9*. No editing alterations were detected in other sites of the transcripts. In maize mitochondria, 18 editing sites exist in *nad3* and 48 editing sites in *nad4* [7]. None of the editing sites in *nad3* are edited 100%, whereas 22 sites in *nad4* are edited at over 90%. Alignments based on the PPR’s sixth 1′-RNA recognition codes show a good agreement with the four completely abolished sites and less so with the partially edited sites [40,41] (Figure 7A). These data imply that editing efficiency at a site may correlate with the binding affinity of DEK48 to that site. If this rationale is correct, we can infer that not all of the edited sites have biological significance, considering the 400 + editing sites (>20% editing level) detected in maize mitochondria [7].

It is difficult to assign the impact on mitochondrial dysfunction to each of the 15 amino acid residue alterations resulting from the absence of DEK48 (Figure 6). We speculate that the editing sites that are completely abolished in *dek48* might have a more severe impact than the others might. Two such sites are in *nad3* and two in nad4. Three of these sites cause alterations of Leu→Pro in Nad3 and Nad4, and one causes Cys→Arg in Nad4 in *dek48*. Proline lacks the amide hydrogen, and the side chain interferes sterically with the backbone of the preceding helix turn, forcing a bend of approximately 30° in the helix axis [45]. Hence, proline interrupts the α-helices and β-sheet structure of proteins. As such, the Leu→Pro changes in *dek48* may severely affect the function of Nad3 and Nad4. Indeed, modeling of the maize Nad3 and Nad4 based on the Cryo-EM structure of the Arabidopsis complex I shows the Nad3-62, -72, and Nad4-126 residues are all in α-helix, whereas Nad4-326 is in the junction between two α-helices [46]. Nad3 and Nad4 are the core subunits of complex I, essential to its assembly and activity [43]. Inferring from the severely disrupted complex I assembly, the alterations of these four amino acid residues may contribute substantially to the disruption of complex I assembly and reduced activity, although we cannot rule out the possible contribution from the other partial amino acid alterations in Nad3 and Nad9 (Figure 6B). Absence of Nad3 or Nad4 inhibits the assembly and activity of mitochondrial complex I [47,48,49,50]. For example, the *dek10* and *dek39* mutants with defects in *nad3* editing showed reduced assembly and activity of complex I [47,48]. Loss of DEK35 and PPR18 specifically impaired the splicing of *nad4* intron 1, leading to a deficiency in *nad4* transcript, resulting in a partially assembled complex I [49,50]. Similarly, the splicing of *nad4* intron 3 was greatly reduced in *dek41/43* mutants, causing the absence of *nad4* transcript, producing sub-complex I [51,52]. These mutants caused the arrested embryo and endosperm development in maize, demonstrating that dysfunction of mitochondria causes seed development arrest. This offers a plausible explanation for the arrested embryogenesis and endosperm development in the *dek48* mutants.

### 3.2. The W Residue in the DYW Triplet Is Essential to the DEK48 Editing Function

DYW-type PPR proteins possess iconic DYW triplet amino acid residues at C-terminus of proteins, and in some proteins, the DYW is changed to DFW, but the W residue is highly conserved. We isolated a point mutation with losing the last W residue in the DYW motif of DEK48 in *dek48-3* (Figure 2). The *dek48-3* kernels are defective, and the editing of all the fifteen sites are impaired similarly as in the *dek48-1* mutant (Figure 6 and Figure S3), indicating that a loss of the W residue in the triplet DYW abolishes the editing function of DEK48. The DYW domain harbors the signature HxE(x)nPCxxC motif found in all deaminases; as such, it is postulated as the deaminase in editing [53]. Recently, it has been proven that the DYW domain possesses cytidine deaminase activity [24,25]. Crystal structure shows that the DYW domain of OTP86 contains a cytidine deaminase fold, a gating domain, and the characteristic DYW motif [26]. The HxE(x)nPCxxC motif is a zinc-binding motif, vital for catalysis and substrate binding [26,54]. The gating domain controls zinc-mediated catalysis sterically, whereas DYW motif is responsible for stabilizing the zinc atom [26]. The W residue in the DYW triplet stabilizes the zinc-binding while maintaining hydrogen bond to the backbone oxygen of Val-919 in OTP86. Indeed, mutation of DYW to DYA failed to complement the *dyw1-1* mutant and showed reduced activity on OTP86^DYW^ [26,54]. Our analysis on *dek48-3* provides strong genetic evidence that the W residue in the DYW triplet is essential to the editing function of DEK48, which is probable in other PPR-DYW proteins.

## 4. Materials and Methods

### 4.1. Plant Materials and Growth Conditions

The *dek48-1* stock (UFMu-06548) was initially isolated from the Maize Genetic Stock Center [34], and *dek48-2* (EMS3-024001) and *dek48-3* (EMS3-0b2fab) from the Maize EMS-induced Mutant Population [35]. *Dek48* transgenic lines were generated by Wimi Biotechnology (Wuhan, China). All maize plants were cultivated either in greenhouse or field at the Shandong University in Qingdao, China. Tobacco (*N. benthamiana*) was grown in growth chambers at 25 °C with a 12-h photoperiod.

### 4.2. Light Microscopy of Cytological Sections

Developing kernels at 9 and 15 days after pollination (DAP) were harvested from the self-pollinated *dek48-1* ears. The kernels were cut, fixed, dehydrated, infiltrated, and embedded as described [36]. The sections were stained with 1% *w*/*v* safranin O and observed using a stereo microscope (Carl-Zeiss, Jena, Germany).

### 4.3. RNA Extraction, RT-PCR, and Quantitative RT-PCR

Total RNA was isolated using the Qiagen Plant RNeasy kit (Qiagen, Germany) and treated with RNase-free DNase I (NEB, Rowley MA, USA) to remove genomic DNA contaminants. Reverse transcription-PCR (RT-PCR) and quantitative real-time PCR (qRT-PCR) were performed according to the manufacturer’s instructions (TransGen, Beijing, China). RNA was normalized against both total RNA and *ZmActin* gene (*GRMZM2G126010*) or *ZmEF1α* (*GRMZM2G153541*). The mitochondrial RNA editing was analyzed as described [8]. Expression of *AOX* genes was analyzed as described [11]. All the primers are listed in Supplementary Table S1.

### 4.4. Subcellular Localization of DEK48

To express DEK48^N258^-GFP fusion proteins, the first 774 bp coding sequence of *Dek48* was cloned and introduced into the binary vector pGWB5 (a gift from prof. Tsuyoshi Nakagawa, Shimane University, Matsue, Japan). The *Agrobacterium tumefaciens* strain EHA105 harboring the fusion construct was infiltrated into tobacco leaves as described [37]. The GFP signals were observed and imaged under a Zeiss LSM 880 confocal microscope (Carl-Zeiss, Jena, Germany) at 24–32 h after infiltration. Mitochondria were labeled with the MitoTracker Red (Thermo Fisher Scientific, Waltham, MA, USA) at a 100 nM concentration.

### 4.5. Phylogenetic Analysis

The amino acid sequences most closely related to DEK48 were extracted from the NCBI (http://www. ncbi. nlm.nih.gov/ (accessed on 18 May 2019)), and the phylogenetic tree was constructed using MEGA6 (https://megasoftware.net/ (accessed on 12 June 2019)) with the neighbor-joining method.

### 4.6. Analysis of Mitochondrial RNA Editing

Total RNA was isolated from *dek48* mutants and WT kernels at 12 DAP and reverse-transcribed as templates. RT-PCR fragments containing the nearly full-length coding sequence of the 35 maize mitochondrion-encoded genes were amplified and sequenced using specific primers as described [8]. RNA editing level of each site was aligned based on the nucleotide peaks in the sequence chromatograms between *dek48* and WT.

### 4.7. Blue Native PAGE and Complex I Activity Assay

Crude mitochondria were extracted from the WT and *dek48-1* kernels at 12 DAP as described [38]. Mitochondrial proteins were solubilized in ACA buffer (0.75 M amino caproic acid, 0.5 mM EDTA, and 50 mM Tris-HCl, pH 7.0) with 2% *w*/*v* final dodecyl maltoside β-DM (Sigma, Santa Clara, CA, USA) and incubated on ice for 30 min. After 20,000 g centrifugation at 4 °C for 15 min, the supernatant was subjected to blue native PAGE and complex I activity assay. A total of 130 μg of crude mitochondrial protein was loaded in 3.5% to 12% BN-PAGE gels and stained with Coomassie brilliant blue (CBB) 250 or incubated in the reaction buffer (0.14 mM NADH, 1.22 mM NBT, and 0.1 M Tris-HCl, pH 7.4) for NADH dehydrogenase activity as described [38]. For immunoblot analysis, crude mitochondrial protein was separated by 12.5% SDS-PAGE, transferred to a PVDF membrane (0.45 mm; Millipore, Burlington, MA, USA), and incubated with primary antibodies against Nad9, Cyt*_C1_*, Cox2, ATPase, and AOX, as described previously [39]. Signals were visualized on X-ray films (Kodak, Tokyo, Japan) using the ECL reagents (Thermo Fisher Scientific, Waltham, MA, USA)

## 5. Conclusions

In this study, we reported a DYW-type PPR protein, DEK48, functioning in the C-to-U RNA editing in maize mitochondria. The mutation of DEK48 arrests embryo and endosperm development, abolishes the C-to-U editing at *nad3*-185, -215 and *nad4*-376, -977 sites, decreases the editing at 11 other sites, and causes reduced assembly and activity of complex I. A point mutation in *dek48-3* causing a deletion of the Tryptophan (W) residue in the DYW motif abolishes the editing function. These results indicate that DEK48 is required for the C-to-U editing in mitochondria and seed development in maize, and they demonstrate a critical role of the W residue in the DYW triplet motif of DEK48 for the C-to-U RNA editing function in vivo.

## Data Availability

Not applicable.

## Supplementary Materials

The following are available online at https://www.mdpi.com/article/10.3390/ijms23063064/s1.
