# Supplementary material for: DEK48 Is Required for RNA Editing at Multiple Mitochondrial Sites and Seed Development in Maize
Source: Int J Mol Sci. 2022 Mar 12;23(6):3064. doi: 10.3390/ijms23063064 (PMC8952262; doi:10.3390/ijms23063064)
Supplement: Supplementary file 1 [file ijms-23-03064-s001.zip › DEK48_sup Figs.pdf]

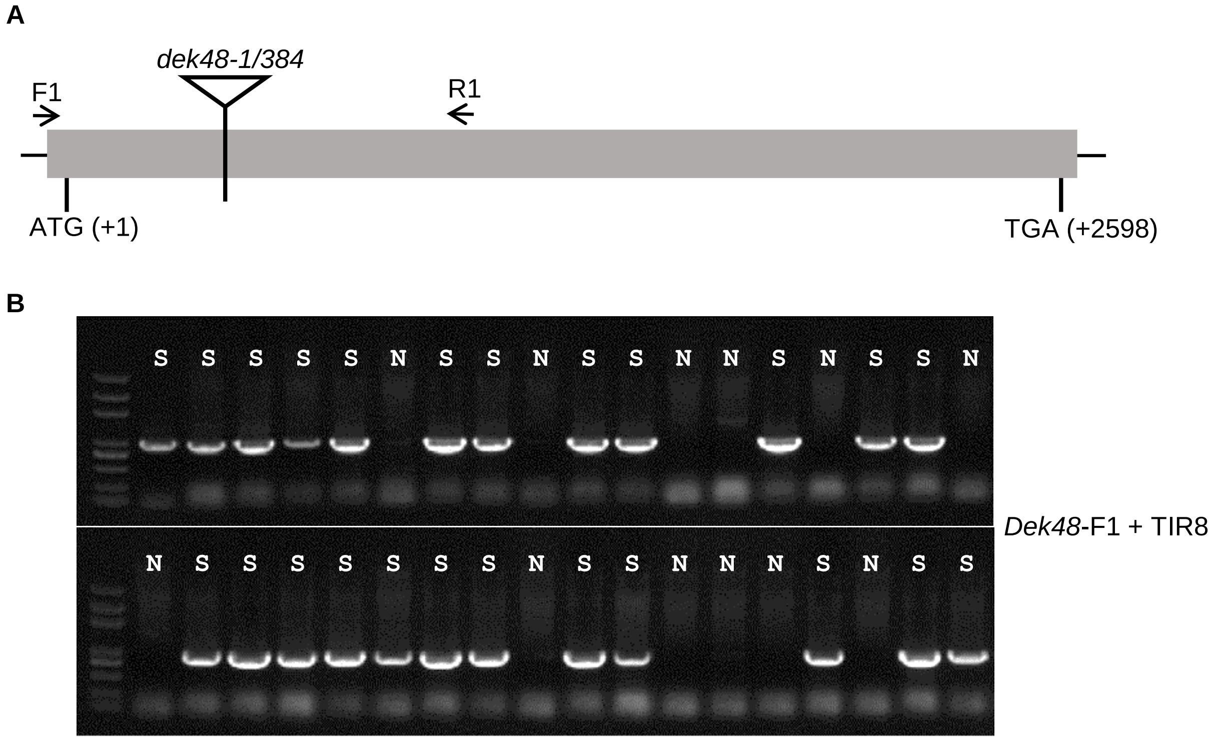

Figure S1. Linkage analysis of the *dek48-1* mutants.

(A) Gene structure of *Dek48* and position of *Mu* insertion. The *Mu* insertion is marked by triangle.

(B) Identification of *Mu* insertion in *Dek48-1* by PCR analysis using the gene specific primer (*Dek48*-F1) and *Mu* TIR8 primer. N: non-segregating (the wild type); S: segregating (heterozygous). The position of primers is indicated in (A).

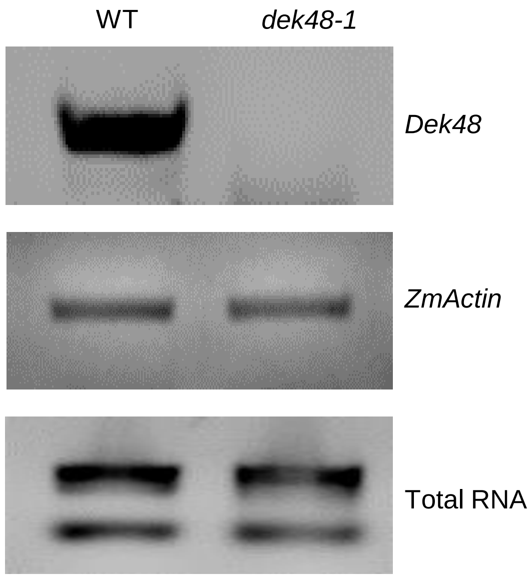

Figure S2. Expression detection of *Dek48* in *dek48-1*.

Normalization was performed against *ZmActin* (*GRMZM2G126010*) and total RNA.

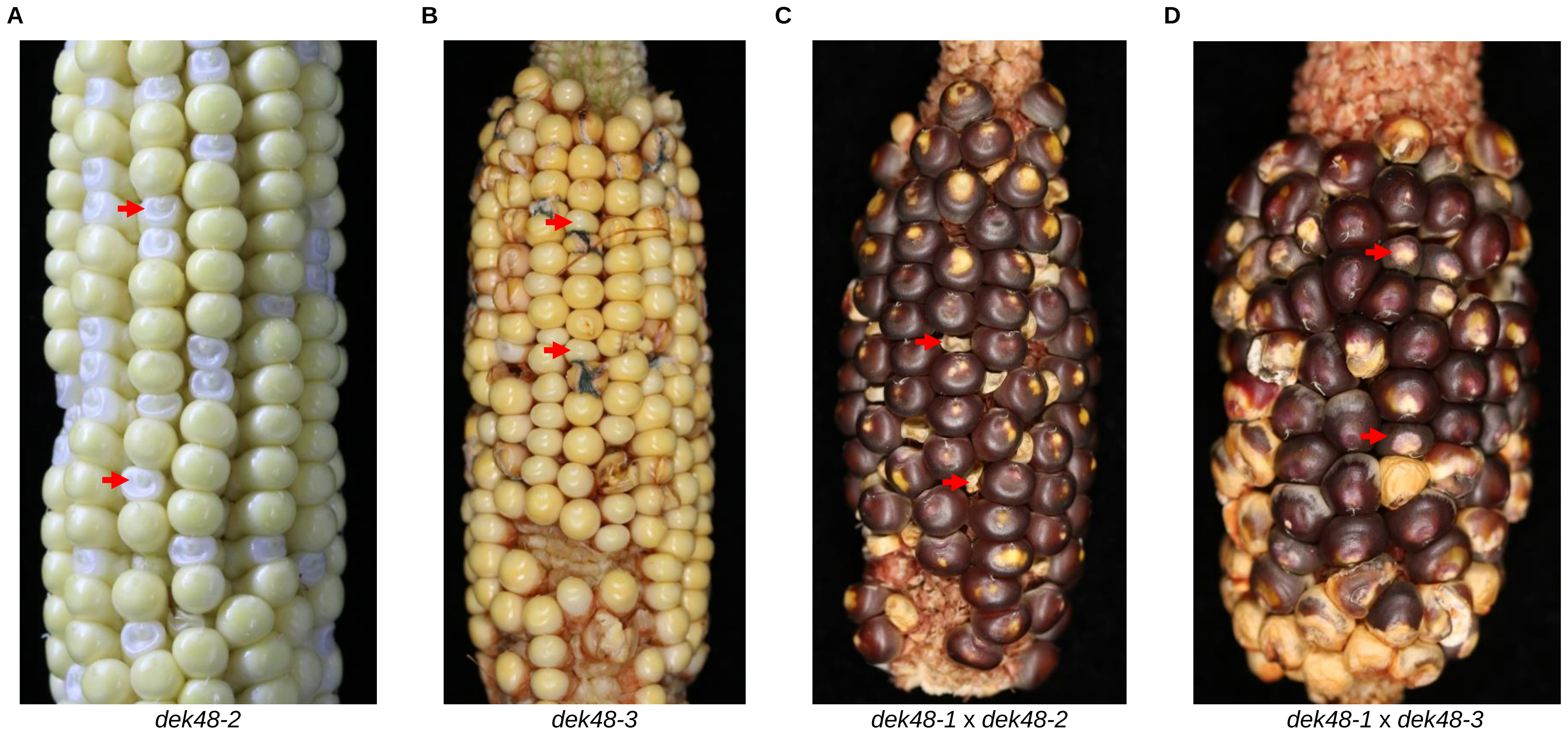

Figure S3. Phenotypes of the *dek48-2* and *dek48-3* alleles and the cross alleles.

(A, B) The selfed ear segregates *dek48-2* at 12 DAP (A) and *dek48-3* at 20 DAP (B). The mutant kernels are indicated by arrows.

(C, D) Kernels phenotype of cross progeny of *dek48-1*×*dek48-2* (C) and *dek48-1*×*dek48-3* (D). The mutant kernels are indicated by arrows.

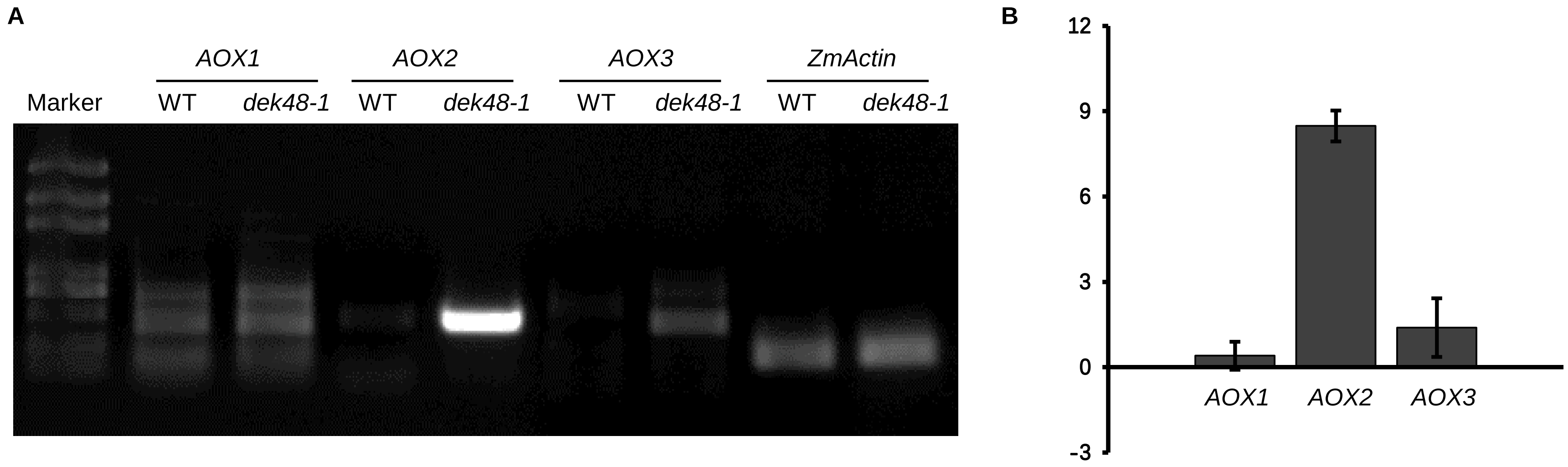

Figure S4. Expression analysis of *AOX* genes in *dek48*.

(A) RT-PCR analysis of *AOX1*, *AOX2*, and *AOX3* expression in WT and *dek48-1*.

(B) qRT-PCR analysis of *AOX1*, *AOX2*, and *AOX3* expression in WT and *dek48-1*. Total RNA was extracted from kernels at 12 DAP. The expression level was normalized against *ZmActin* (*GRMZM2G126010*).
