# Supplementary material for: DEK48 Is Required for RNA Editing at Multiple Mitochondrial Sites and Seed Development in Maize
Source: Int J Mol Sci. 2022 Mar 12;23(6):3064. doi: 10.3390/ijms23063064 (PMC8952262; doi:10.3390/ijms23063064)
Supplement: Supplementary file 1 [file ijms-23-03064-s001.zip › DEK48_Sup Table.pdf]

Primers used in the DEK48 study.

Primer name	Primer sequences (5'-3')	Application
Dek48-F1	CCTACACCAGATCGGAAGTCCC	Genotype *dek48-1* mutant
Dek48-R1	GCACTCTGGAGATTGTCGCACT	
TIR8a	CGCCTCCATTTCGTCGAATCCCCTS	
TIR8b	CGCCTCCATTTCGTCGAATCCSCTT	
TIR8c	SGCCTCCATTTCGTCGAATCCCKT	
TIR8d	CGCCTCCATTTCGTCGAATCACCTC	
Dek48-F2	CCTCCATGCCTACCTCTCCTG	Genotype *dek48-2 mutant*
Dek48-R2	TCCAAGCCACATTGTTACGGT	
Dek48-F3	AATGCTGCTGCCCCTTTGACT	Genotype *dek48-3 mutant*
Dek48-R3	CTCCGAGATTCACCACAGCGA	
Dek48-F4	CACCATGGCGGTGACGCAGGCGTTT	*Dek48* cloning into pEntry vector for subcellular localization
Dek48-R4	AACCATATCAAGGGCTTCT	
Dek48-F5	GGAACCTACCGCACATTGATC	RT-PCR and qRT-PCR analysis of *Dek48* gene
Dek48-R5	CAGACAAAGTGAATGTAGTTGCCTC	
Dek48-F6	TTTTGGATCCATGGCGGTGAC	*Dek48* cloning into pUNTF vector for overexpression
Dek48-R6	TTTTCCCGGGTCACCAGTAATC	
Ubi-F	CTTGGATGATGGCATATGCAGC	Genotype *Dek48*-OE and *Dek48*-Com
ZmActin-F1	TAGTTGAGAATGGCTGACGAGG	Internal normalization
ZmActin-R1	ATCTTCAGGCGAAACACGGAGC	
ZmEF1α-F	TCTCAAGAACGGTGATGCTG	
ZmEF1α-R	TGGGTCCTTCTTCTCCACAC	
